# A Course of Lectures on Dental Physiology and Surgery.

**Published:** 1848-07

**Authors:** John Tomes

**Affiliations:** Surgeon Dentist to the Hospital.


THE AMERICAN

### Journal of Dental Science.

Vol. VIII.] JULY, 1848. [No. 4.

### ARTICLE I.

*A Course of Lectures on Dental Physiology and Surgery, delivered at the Middlesex Hospital School.* By JOHN TOMES, ESQ., Surgeon Dentist to the Hospital.

(Continued from page 246.)

### LECTURE XIII.

DISEASES OF THE DENTAL PULP—GRANULATION OR POLYPUS OF THE PULP—ITS NATURE AND TREATMENT—RECESSION OF THE PULP—DISEASES OF THE DENTAL PERIOSTEUM—INFLAMMATION, ACUTE AND GENERAL; CHRONIC AND PARTIAL—SYMPTOMS AND TREATMENT

*Polypus, or Granulation of the Dental Pulp.*—After partial destruction of the crown, and, especially, of the masticating surface of a tooth, and the consequent opening of the central cavity, the pulp instead of perishing may become the seat of morbid growth. Rising out of the damaged pulp cavity, you may find a small rounded, smooth-surfaced, vascular tumor, which bleeds from the slightest touch, and constantly emits a most fetid smell; even the blood that flows from it stinks. The new tissue, as compared with the pulp itself, is endowed with but a low degree of sensibility. Slight pressure, or even superficial laceration, produces but little pain.

The size to which dental polypus may grow is subject to considerable variety. Sometimes the tumor is small, resembling in size and appearance a large granulation; at others, so large as to cover over and completely hide the wreck of the tooth from the centre of which it has sprung. In the latter case the overhanging portion of the tumor may unite with the gum, and thus bury the tooth in vascular tissue.

Although polypus of the pulp is not necessarily attended with pain, yet should the new tissue be attacked with inflammation or ulceration, it then becomes extremely sensitive and painful.

*Treatment.*—The only radical cure is the extraction of the tooth in which the disease has its site. Should, however, your patient object to this measure, (and we cannot always make patients do as we would have them,) the tumor may be cut off as low down in the fang as can be reached, and its future growth kept back by the application of astringents. Tannin applied frequently will answer the purpose.

*Recession of the Dental Pulp.*—I have already told you that the pulp, under certain conditions, gradually wastes in volume and at last disappears. But there are some cases in which it is gone before we suspect the process of absorption to have commenced. To these I will draw your attention but for a moment, and then we shall have done with the diseases of this small, but sometimes most troublesome, organ.

Patients are occasionally met with who state that their teeth, one after another, have gradually and painlessly decayed away; that they have never had an hour's toothache or even tenderness of teeth, though now nothing but stumps are left in the mouth: in what manner, and when the pulp has been removed, we are unable to tell. All that we know is, that it has gone, and all that it is necessary to know is, what should be done with the tooth so circumstanced.

*Diseases of the Dental Periosteum.*—By a natural transition we pass from diseases of the dental pulp to diseases of the dental periosteum. Inflammation and its consequences, modified variously, either by the producing cause or by constitutional peculiarity, or both, form the principal diseases of this appendage of the teeth. To the investigation of these we must now lend our attention, and first of all to inflammation so located. The character of the morbid action may be that of ordinary inflammation, or it may be modified by some pre-existing state of the system. The inflammation may take a rheumatic or a strumous type, in patients afflicted with rheumatism or struma, or it may take its character from the past or present influence of mercury on the system. Then, again, the whole, or only a part of the periosteum may be affected.

It will be best for me to take, first, inflammation of the dental periosteum in its most common, and hence most important forms; namely, active and chronic, and afterwards the various modifications of the disease. Before going further, however, I wish it to be understood that all those cases which terminate in the formation of pus in the alveolar cavity will be placed under the head of *active inflammation*, while the term *chronic inflammation* will be reserved for those in which the periosteum becomes thickened, the tooth loosened, and, the disease in that condition, if left to itself, smoulders on, till at last the tooth drops out. Of course there are many cases of a mixed character, which, in their various stages, belong as much to the one as to the other of these arbitrary divisions; but, with a knowledge of the two extremes, we are prepared for a medium case. Neither is it strictly correct to consider all cases of alveolar abscess as the result of active inflammation, seeing that the disease is often slow, and comparatively painless. Yet we must, for the convenience of description, have some division and arrangement of the subject, and the one I have proposed will suit our present purpose.

*Active Inflammation of the Dental Periosteum.*—The inflammatory action usually sets in with feelings of slight uneasiness and tension, which sensations are attended with a strong desire to press by the opposing teeth, or to shake with the fingers, the affected tooth in its socket. Slight steady pressure of the fang into the jaw gives relief so long as it is maintained, but the uneasiness returns on its withdrawal. At the same time vioent and irregular pressure will produce pain. The uneasiness is soon followed by a dull heavy pain, and the tooth feels to be too ong. The desire to push the tooth about continues, till disease has rendered the parts so tender that pressure can no longer be borne, and even the mouth cannot be firmly closed without pain. The tooth seems still to lengthen, and on an attempt to move it from side to side feels slightly loose. These latter symptoms obviously depend on swelling of the periosteum, and consequent lifting of the tooth in its socket. Early in the complaint there is tenderness and swelling of the gum, and generally of the external gum, opposite the fangs, whose periosteum is affected. In addition to this latter symptom, and often prior to its appearance, the free edge of the gum assumes a deep red color, without either pain, tenderness, or scarcely any swelling. The neck of the tooth appears encircled with a well defined red ring. I am not prepared to say that this symptom is always present, though, undoubtedly, it is seldom absent in the earlier stage; but as the disease advances this appearance is lost in the general inflammation of the gum. The pain becomes more severe, but still preserves its heavy wearing character, and, though not always constant, is seldom absent for many hours. These symptoms lasting, the part of the periosteum most highly inflamed, becomes detached from the surface of the fang. The most common seat of this denudation is around the apex of the fang. Into the interval thus formed pus is poured from the separated surface of the periosteum. The fang at this part loses its vitality, and is bathed in pus, which latter gradually increases in quantity, room being made in the alveolus for the dilation of the abscess at the expense of the bone. In some of the specimens on the table you will perceive that the bone is closely fitted to the neck of the fang, while about the apex the alveolus is hollowed out into a round cavity large enough to hold a pea. With the formation of pus a process is established for effecting its escape. Either the periosteum becomes detached through the whole length of the fang, and the matter is discharged at the neck of the tooth, or, what is much more common, a hole is formed in the wall of the alveolus, through which the pus gets into the gum. We have then what is commonly called a gum-boil, an abscess in the gum communicating by a small hole through the bone with an abscess in the alveolus. If left to itself the abscess will work its way to the surface, and the contents will be discharged into the mouth. The symptoms now abate, the pain ceases, and the swelling lessens, but a minute fistulous opening in the gum remains, through which a small amount of discharge habitually escapes. The inner surface or coat of the alveolar abscess I have told you is formed by periosteal membrane detached by inflammation, and stretched out from the fang by the gradual interposition of pus. When the abscess breaks, and the contents readily escape, the inner membrane contracts and closes upon, but never unites with, the denuded surface of the fang. While the inner surface of the abscess contracts upon the fang the outer parts thicken and occupy the space which would otherwise be left between the abscess and the walls of the expanded alveolus. If a tooth be drawn after this has occurred, the coats of the abscess often come out adherent to the fang in the form of a fleshy appendage, and the tooth is said to have a fungous growth from the fang. Reunion between dental tissue and periosteum separated by disease, I believe never occurs. On the contrary, the denuded and consequently dead tooth substance, when in contact with, proves a constant source of irritation to the living and already diseased periosteum; hence, the lasting discharge and consequent fistulous opening. The quantity and the quality of the continued secretion varies. Sometimes it is almost imperceptible and serous; at other times it is abundant and purulent; varying in different individuals, and in the same individual at different times.

Such is the course which active inflammation of the dental periosteum usually pursues when left to itself.

The disease may, however, assume a much more active character; all the symptoms may be aggravated, and, in addition to which, there may be considerable symptomatic fever.

The following case occurred in the person of a medical practitioner, and will give you a good idea of the severity the disease may assume:

CASE.—T. S., age 30. In 1840, the crown of the left central incisor was broken off by a blow from a cricket ball; I refixed the crown by a pivot. In 1844, the crown had become discolored, and was replaced by the crown of a mineral tooth. The dentist who performed the operation found it necessary to increase the length of the hole in the stump, for the reception of the pivot. This was done on the 8th of October; on the morning of the 10th, slight pain was felt about the root of the tooth. Towards evening, the pain had greatly increased; the pivot was then removed. The night was passed in pain, and without sleep, and in the morning a fit of cold shivers was followed by heat, and terminated in profuse perspiration. 11th.—The pain still increasing, and attended by œdematous swelling of the face; added to which, there was great symptomatic fever. Pulse 130, full and hard, and accompanied by headache; twenty ounces of blood were taken from the arm, and salines were administered. Fomentations were applied to the face, and a dose of Dover's powder given at bed-time. 12th—Considerable and extending inflammation of the mouth near the pivoted tooth; more swelling of the face; headache rather less severe; six leeches were applied over the gums; blood taken yesterday rather buffy. 13th.—Swelling and pain much the same; a small abscess formed in the roof of the mouth, near the tooth. This was opened. The fomentations were continued, and the Dover's powder repeated at bed-time. 14th.—The abscess in the roof of the mouth was again opened, and a considerable quantity of purulent matter let out; the inflammation, pain and swelling much the same; six more leeches were applied, followed by fomentation and poultice; the Dover's powder repeated at night. 15th.—Symptoms no better; three more leeches were applied to the gums, and the fomentations were continued. 16th.—From this time the symptoms gradually abated, but left a small abscess over the stump, from which a considerable quantity of pus escaped.

On the 5th of November he applied to me. There was still pain in the stump; the gum was swollen, and pus escaped from an opening opposite the end of the fang, which, on removal, was found to be discolored, and evidently dead. The operation was followed by the escape of a quantity of unhealthy pus. From this moment no further inconvenience was felt; the gum healed, and the whole mouth returned to a state of health.

The stump was not transfixed by the second operation of pivoting; I mention this because I have seen several cases where a similar course of events have followed the transfixture of the fang in the operation of pivoting.

An equally instructive case, in which the disease was cut short, occurred in the son of the hospital carpenter, a lad of 14 years of age. He was attacked with severe pain in the canine of the upper jaw on the right side. The tooth was perfectly sound. The pain increased in severity for six-and-thirty hours, and the tooth then felt too long, and was greatly in the way when he closed his mouth, from the pain that was occasioned whenever it was touched by the opposing teeth. There was considerable headache, thirst, and a loaded tongue, and the boy said, "he felt almost light-headed at times." I removed the tooth, and found the fang everywhere coated with thickened and inflamed periosteum. The pain ceased with the smart of the operation, and there was no return of suffering. Had this tooth been allowed to remain, the case would, no doubt, have followed a similar course to that I just now described. An abscess would have formed in the alveolus, and, after more or less suffering, opened on the surface of the gum. The specimen is before you, and you may contrast for yourselves the diseased of this, with the healthy periosteum of other teeth.

### LECTURE XIV.

ABNORMAL CONDITIONS OF THE ALVEOLI—NECROSIS—ALVEOLAR EXOSTOSIS—ABSORPTION OF THE ALVEOLI.

DISEASES OF THE GUMS—INFLAMMATION, ACUTE AND CHRONIC, OF THE GUMS—ULCERATION OF THE GUMS—TUMORS OF THE GUMS—EPULIS—POLYPUS—VASCULAR TUMORS OF THE GUMS—BLUE GUM—SALIVARY CALCULI, OR TARTAR.

### *Abnormal Conditions of the Alveoli.*

I SHALL pass over the symptoms and treatment of those diseases of the jaw which are common to all bones, because they are treated of in your surgical lectures. Those abnormal conditions, however, which occur in connection with, and exercise an influence on the teeth, I shall notice so far as the treatment affects the teeth.

*Necrosis of the Alveoli.*—Death of a portion of the alveolar arch is far from uncommon. The greater part of the jaw may be necrosed or the disease may be confined to the socket of a single tooth, or even a portion of the socket. It may occur at any period of life, and to either jaw, but is more frequent in the under jaw. Sometimes we find necrosis of the alveoli in quite young children. Two or three of the sockets of the temporary teeth are lost, including also the cells containing the partially developed permanent teeth. Again, we find the same disease in old people, affecting perhaps the sockets of their few remaining teeth. An old man of sixty, recently in attendance at the hospital, lost the central incisores, with their alveoli, by necrosis; and these were the last of his teeth.

The symptoms of necrosis of the alveoli are similar to those indicative of the same disease occurring in any other bone, and the treatment must also be similar. Mechanical injuries of the jaw are frequently productive of necrosis. The extraction of diseased teeth unskilfully performed is a frequent cause of death of a small portion of the jaw; and this catastrophe may, and sometimes does, follow the drawing of a tooth, however dexterously managed. In the great majority of cases, however, diseased teeth play an important part in the production of the disease, and have, therefore, to be considered in the treatment.

The dental pulp becomes exposed by caries or injury of the crown, inflames, and the inflammation extends to the dento-alveolar periosteum, and from thence to the jaw, and thus produces necrosis.

The teeth situated over the dead bone loosen and drop out, or much more frequently are removed by the surgeon. There can be no doubt of the propriety of removing the tooth that has occasioned the disease, but it becomes a question whether sound teeth loosened in necrosis should be at once removed. In the following case, read at the Medico-Chirurgical Society, by Mr. Sharp, the teeth were loosened by necrosis of the jaw, but afterwards became quite firm and useful, and, as it would seem, by the formation of the new alveoli. The sequestrum was exhibited, and figured for the Transactions. I have since had an opportunity of examining the sequestrum, and my friend Mr. de Morgan has been kind enough on this, as on many previous occasions, to make a drawing of the specimen a little more favorable for showing the alveoli than that contained in the Transactions. The case was reported as follows:

"Esther Watson, aged 20, consulted me, on the 8th of September, 1842, for an extensive necrosis of the lower jaw, with ulceration of the integuments under the chin. The mouth and lower part of the face, from the tumefaction, the ulcers, and the fetid discharge, presented a very disagreeable appearance. It had commenced with toothache about six months before, the pain being followed by an inflamed swelling, the swelling by abscess, and the abscess by ulceration. No effectual treatment had been adopted to arrest the progress of a very painful disease.

"It immediately occurred to me that a fungus arising out of the fang of the decaying tooth had been the origin of the mischief; and my first object was to remove this by extracting the tooth. The posterior bicuspid of the left side of the lower jaw was accordingly drawn, and a small fungous growth was found attached to one of its fangs. On examining the jaw with a probe through the ulcerated openings, a large extent of denuded bone was felt, but it appeared to be firmly attached. My further attempts were therefore limited to prescribing an alum gargle to be freely used to cleanse the mouth, and zinc ointment to be applied externally.

"By these means the *comfort* of the patient was greatly promoted, and time was allowed for the process of separation to advance: they were continued, with the occasional application of a poultice, for three months.

"On the 13th of December, it being evident that the dead bone was completely detached from the living, the existing openings under the chin were thrown into one, and somewhat enlarged by two small incisions. The bone was taken hold of by a pair of forceps, and immediately, without pain or hæmorrhage, extracted. The portion removed amounted to about two-thirds of the entire lower jaw, and contained several of the alveolar processes.

"A slight dressing was applied to the wound, and, on desiring the patient to open her mouth, to my very agreeable surprise I found the entire set of excellent teeth (with the single exception of the one I had extracted) perfectly fast, and in their proper places.

"On the 5th of Jan'y, 1843, (in three weeks) the wound was very nearly healed, and the face had now recovered a perfectly natural appearance, having lost the swelling and inflammation which had disfigured it for so many months. She then returned into the country, and, as far as I have been able to learn, has since continued quite well."—*Medico-Chirurgical Transactions*, vol. xxvii, p. 432.

I am not aware that a similar case has been since reported. We have, however, had an out-patient admitted under Mr. de Morgan, whose case, so far as it was seen, followed very closely to that described by Mr. Sharp. The patient was an Irish woman of about five-and-thirty. She stated that she was attacked with violent toothache, at first confined to one tooth, that the pain afterwards extended to the jaws, and that the jaw became swollen. When she came to the hospital there was considerable thickening of the front and right side of the lower jaw, with three fistulous openings in the gum, through which dead bone could be felt. The sequestrum, which was not detached, seemed to extend from the front to the side of the jaw as far back as the second molar, over which part the teeth were sound, but very loose. Pressure on the teeth produced pain; but when moved about, the fangs did not grate upon denuded bone. General and local treatment was adopted for subduing the inflammation, and the loose teeth were allowed to remain. At the end of a fortnight the pain and inflammation had subsided, and the teeth had become more firm. Two months afterwards the teeth had become comparatively firm, and the swelling about the jaw had greatly subsided, but the dead had not completely separated from the living bone. The woman now thought very lightly of her malady, and could not be persuaded that there was any disease left worth her attention; and from that time she has been lost sight of.

Then, as regards teeth loosened by necrosis of the jaw and alveoli, it will be well to let them remain if they are sound, and are not, so far as we can tell, stripped of their periosteum, and do not occasion irritation. Much will be gained if they are by nature refixed in new sockets; and though the probabilities are greatly against the occurrence of this event, yet we need risk nothing in taking what chance there may be. Wherever there is fibrous tissue there may be formation of bone; and it is possible that, if the dento-alveolar periosteum escapes destruction, it may contribute to form new alveoli.

*Alveolar Exostosis.*—After a tooth has been pulled out, the vacated socket is gradually filled up from the bottom by bone. This growth of bone in the socket sometimes takes place without the previous removal of the tooth, and thus gradually forces the tooth out of its natural position, or out of the jaw. This happens more commonly to the central incisores of the upper jaw than to any other teeth.

You may sometimes see people in whom one front tooth looks longer than its fellow, the one having been protruded by exostosis in the socket. In other cases, again, the teeth are forced apart by thickening of that portion of the alveolus that passes between them, or they may be pressed outwards or inwards by osseous thickening of the outer or inner wall of the socket. The fangs of the displaced teeth are sometimes also shortened by absorption.

The disease will have its way in spite of treatment: hence in most cases treatment is but an addition of evil to that consequent on the malady. Eventually the tooth is forced out, or becomes so much displaced, or discolored from exposure of the fang, that its removal is necessitated.

*Absorption of the Alveoli.*—In advanced age the alveoli become absorbed, and the teeth, however sound in themselves, fall out. The process commences on the edge, and gradually advances towards the jaw, till the whole of the sockets are removed. This, which is a natural occurrence in the old, constitutes a disease when it happens to the young or middle-aged, and produces premature age in the jaws. Frequent or long-continued salivation, scurvy, purpura, are a few of the many constitutional conditions that lead, directly or indirectly, by producing increased vascularity of the gums, to premature absorption of the alveoli; while the use of a very hard tooth-brush, the accumulation of tartar ligatures, whether of silk or metal, round the necks of the teeth and in contact with the gums, by inducing irritation in the edge of the gums, lead to a like result.

In cases arising from any of these conditions, the disease may be arrested by removing the cause, and afterwards employing astringents to the gum.

Premature alveolar absorption sometimes, however, occurs without any accompanying disease of the gums, teeth, or periosteum, and in such case is not favorably influenced by treatment. This form you will find running through the various members of a family, and descending from father to son. The sockets of many or only a few teeth may waste away, while others remain unaffected; those of the front teeth being most frequently absorbed.

The patient should be enjoined to scrupulously avoid any sources of irritation of the gum, and to use a very soft tooth-brush, and to prevent the accumulation of tartar on the necks of the teeth.

### *Diseases of the Gums.*

*Acute Inflammation of the Gums.*—This disease is of rather rare occurrence, except as the consequence of specific agents administered for the cure of disease.

There are, however, a few cases on record of spontaneous salivation, in which the gums have been highly inflamed. I saw, a few months since, a case in which the gums of the upper jaw, and especially of the anterior part of the jaw, had become highly inflamed without any assignable cause; the pain in the mouth was great, and the flow of saliva excessive; the disease yielded to free scarification, astringents, and occasional aperients.

In salivation produced by mercury, the effect is first discernible upon the gums. Some hours previous to the coming of the metallic taste, and to the fetor of the breath, and also to the soreness and discomfort which mark the influence of mercury on the system, the gums show indications that these conditions are about to appear—in fact, that the patient will in a few hours be salivated. The state of the gum I am about to describe is, in fact, a premonitory sign of ptyalism, for should it appear, and the mercury be immediately discontinued, yet salivation will come on. The sign is this: the adherent portion of the mucous membrane of the gums assumes an opaque white color, contrasting strongly with the non-adherent portion, which preserves its natural hue or becomes more red. The free edge of the gums is movable, but that part which lies over the edge of the alveoli is firmly tied down to the periosteum; and as the edges of the alveoli present a festooned line, so the whitened mucous membrane presents in a corresponding festooned line. Again, where the mucous membrane is loosely reflected from the gum to the cheek, the natural color is preserved. The whiteness of gum is produced by an increased secretion of epithelium, which from being thicker and more opaque renders the color given by the vessels to the subjacent tissue less apparent.

The surface of the mucous membrane when deprived of epithelium is studded over with innumerable small conical elevations, or papillæ. The thickened epithelium is readily rubbed off the tops of the papillæ, while it retains its full thickness in the hollows between them; thence, if closely inspected, the gums will not be seen to present a uniform white hue, but a mottled aspect; and this because the epithelium is thin over the papillæ and thick between them, and therefore more color will show through at one part than at another.

With the increased thickness there is a decrease of tenacity between the scales that form the epithelium, for the surface may be much more readily rubbed off than when in its natural state.

This curious and useful premonitory sign of coming ptyalism was, I believe, first noticed, and its value pointed out, by Mr. Corfe; at all events, he first of all drew my attention to the fact, and I am not aware that it has been described by any author. Since, however, Mr. Corfe mentioned the result of his observations as to the constancy of the sign, I have verified for myself its presence in all cases of salivation that have come under my notice, and from these I have written the foregoing account.

If you would make use of this indication in your practice, it will be necessary that you should carefully note the state of the gums at the time the mercurial treatment is commenced, for it is quite possible that other agents may produce a similar state of gum, and that such may exist previous to the exhibition of mercury.

*Chronic Inflammation of the Gums.*—This form of disease is very common in the middle and later periods of life, and when once established is apt to prove obstinate.

The surface of the gums becomes minutely modulated, and the secretion of epithelium increased; the papillæ are increased in prominence, while the substance of the gum is generally thickened, and the edges about the teeth thick and round. The disease may be confined to the gum, about two or three teeth, or may extend over the whole mouth; the extent depending upon whether the cause be local or general.

The amount of pain consequent on this malady is subject to considerable variety, both in degree and character. In some cases but little uneasiness is complained of unless when eating, while in others the mouth is seldom free from pain. Then, again, in others it comes and goes irregularly, or sometimes regularly; the pain may come on every evening towards bed-time, and last for several hours. In any case the severity is usually increased in damp weather.

Chronic inflammation of the gums, if allowed to pursue its own course, may lead to one of two very opposite results; the alveoli may become absorbed, or they may become thickened and spongy. In either case the teeth eventually are loosened, from the diseased state of the gums spreading to the dental periosteum.

This form of disorder is very commonly the result of indigestion, when general; when confined to part of the mouth it follows pre-existing disease of the dental periosteum, of stumps, or diseased teeth.

*Treatment.*—The cause should be removed—the affected gum should be from time to time lightly scarified with a sharp lancet, much in the manner that granular conjunctiva is scarified; astringents should be frequently applied, and of these tannin is the best: this powder should be rubbed with the finger twice or thrice a day over the affected part. Teeth that have been loosened by inflammation of the gums tend to keep up the disease by their mobility; the patient may not, however, consent to their removal, especially if the front teeth are the ones implicated. In this kind of case I have seen great benefit follow the use of compound spirits of horse-radish, and also spirits of scurvy-grass; they should be applied to the affected gum, three or four times a day, with a bit of soft sponge.

There is a singular modification of chronic inflammation of the gums, in which the gum, instead of thickening and becoming irregular on the surface, seems rather to decrease in size, assumes a very smooth and polished surface, and mottled aspect; the disease often extends over the surface of the hard palate, and is attended with acute intermittent pain; the pain may be confined to one side of the mouth, or even to half of the upper jaw; it very commonly comes on in the evening, and keeps the patient awake half the night. The cases of this complaint that have come under my notice have been confined to poor middle-aged females, in whom menstruation was becoming irregular, or had altogether ceased; and they have always been cured by the regular use of mild aperients. I have usually given a small dose of sulphate and carbonate of magnesia twice a day, and at the end of a week or nine days the pain in the gums has ceased, and that structure has assumed its healthy appearance.

*Ulceration of the Gums.*—The gums are frequently the seat of ulceration: a small, round, excavated ulcer appears, of a yellowish-white color, very painful when touched, and hence troublesome. It may occur in any part of the gums, but when it is situated between the teeth its existence is not readily recognized: hence patients have had sound teeth removed to relieve pain which was, in fact, not in the tooth, but in the interdental gum. Ulcers of this kind not uncommonly make their appearance on tumors of the gum, in situations where they cannot be seen, and so give much trouble.

There is one unfailing and almost instantaneous remedy—this is, nitric acid; the ulcer should be touched with the acid diluted with an equal part of water; a camel's hair pencil is the best instrument for applying the remedy.

I told you in a previous lecture that polypus of the pulp sometimes attains a considerable size, spreads over the edges of the fang from which it has sprung, and unites with the surrounding gum, thus burying the stump in the new tissue. When in this state, spreading ulceration sometimes attacks the surface of the new texture, and would destroy it but that it is constantly growing at the base, and thus affords material for the ulcerative process to continue. I have seen one case of this nature, in which the surface of the ulcer looked like malignant disease; the surrounding parts had not, however, the induration peculiar to that frightful malady. The stump was felt with a sharp steel probe, and removed, and then the gum speedily returned to a healthy state.

*Tumors of the Gums.*—Tumors occasionally grow up in the gums, having originated in the vessels or vascular canals of the bone, in the periosteum, or in the substance of the gum itself.

When arising from either of the former tissues, the tumor is termed *epulis;* while those springing from the gum itself, being similar in structure to the gum, are usually called *polypus*, or *granulation* of the gum.

*Epulis.*—This species of tumor, itself fibrous, is developed in the fibrous tissue about the bone, from which it continues to grow, and presses before it the tissues of the gum; hence in external appearance it resembles in color, and generally in surface the gum; or the surface may be rough, and then it looks like a wart on the gum. The growth is generally slow, but gradually progressive, and usually unattended with pain, and the tumor, although tolerably vascular, is not prone to bleed.

The surface of the bone on which the tumor rests becomes more or less affected, the degree being proportionate to the duration and magnitude of the tumor. The Haversion canals in this part become much dilated, and they contain portions of the the tumor; for should it be excised down to the surface of the bone, but the canals left, the tumor speedily reappears; while if the affected bone be also excised the disease does not reappear.

Osseous tissue may be increased in quantity by a diminution in the calibre of the Haversian canals, or by an addition to the surface; or it may be diminished in quantity by the dilatations of the Haversian canals, or it may die, but in neither case does the tissue itself undergo any change; it is but a matter of *more* or *less*. Hence when epulis seems to grow from, or out of, the bone, it does not belong to the osseous tissue, but to the contents of the Haversian canals that traverse that tissue.

Osseous spiculæ not uncommonly shoot from the surface of the jaw into the tumor, and in some cases isolated nodules of bone are found in its substance; in the latter feature epulis strongly resembles fibrous tumors of the uterus.

Epulis is strictly a fibro-cellular tumor, composed of interlacing fibres, mingled with nucleated cells, in every stage of advancement towards the formation of fibres. This disease may form in any part of the gums, but it most commonly springs up in that part which passes between the teeth.

Your patients will tell you that they first of all found a small lump on the gum, but that it gave no pain, and hence, for a while, did not engage the attention. As it increased in size, however, the teeth at its base loosened, were separated from each other, and got out of place. As the growth advances, the tumor becomes softer, more vascular, is indented by the teeth, and subject to ulceration, in which case it also becomes painful; sometimes it will assume very much the appearance of malignant disease. If a tumor of this nature be left to itself, it may gradually attain a considerable size, and lead to great displacement of neighboring parts, and disfiguration of the face.

When the disease has become formidable from its size or character, the treatment falls to the province of the operative surgeon; and to surgical writers I must refer you for a more detailed account of the disease and its treatment, but I would especially recommend your perusal of a clinical lecture on the subject, by Mr. Cæsar Hawkins, published in the Medical Gazette about eighteen months since.

Whatever be the extent of the tumor, extirpation and subsequent excision, or destruction by escharotics, of the surface of the bone from the canals of which it has grown, is the only sure manner of removing this otherwise obstinate disease.

*Polypus, or Fungus of the Gum.*—This disease is essentially hypertrophy of the gum, arising from mechanical irritation. If a tooth decay away on one side to below the level of the gum, leaving a sharp margin in contact with the gum, a tumor frequently forms from the gum, spreads into, and partially fills up, the hole in the tooth, or the vacancy between the two decaying teeth. The tumor is usually composed of dense fibrous tissue, covered with epithelium; is almost insensible, unless ulcerated, when it becomes very painful. If the tumor be removed it will grow again and again, unless the bad tooth is removed, and then it will speedily disappear. These tumors show on section an undulating surfaced fibro-cellular tissue, covered by a thick layer of epithelium.

Caries sometimes attacks that surface of a tooth which lies in contact with a neighboring tooth, in which case, before an instrument can be introduced to excise the softened dentine, a portion of the tooth must be cut away; and it is sometimes necessary, before the operation of plugging is completed, to cut away portions of the tooth as low down as where the gum becomes attached. This operation is sometimes followed by irritation and slight polypus of gum. The new growth is highly sensible to pressure, and if food is forced by mastication on such a part, pain resembling toothache is felt. A small ulcer will sometimes form in this situation, and the pain simulate toothache so closely, that the patient desires that the tooth may be drawn; and I have seen teeth removed in such cases. The best application for the relief of this troublesome state of the gum is sulphate of copper applied every day or two. I have found a little powder, introduced on a quill and dropped on the part, is the most convenient mode of application; the relief is often instantaneous. The remedy should be applied some little time after the gum has ceased to give trouble. Other escharotics would no doubt answer equally well. If a sharp edge of tooth, or of projecting stopping, produce the irritation, no local application will be useful until these sources of mischief are removed by the file or otherwise. A similar state of gum is sometimes produced by the presence of a jagged piece of tartar, in which case the disease subsides with the removal of the cause.

*Vascular Tumors of the Gums.*—The gums are occasionally the seat of vascular tumors closely resembling in physical characters ordinary nævus. I have met with two cases of this disease: one of these I will describe. A female patient, of about 25 years of age, stated that some time past she observed a small red pimple between the front teeth of the upper jaw, which bled every time she cleaned her teeth; that it gradually grew and spread on the surface of the gum, both in front and behind the teeth, and that with its increase of bulk the front teeth had separated and loosened; and that at times she suffered a good deal of pain in and about the tumor, which had become tender on pressure and very liable to bleed.

When the patient first came to me, the central teeth were separated about the eighth of an inch, were loose, and very tender. The tumor occupied the interval, and reached half way down towards the edges, and had spread itself over the gum immediately in front of the teeth for about half an inch, and covered a similar space on the palate behind the teeth. It was of a bright scarlet color, and soft in texture, and could by steady uniform pressure be deprived of color, and reduced to the level of the surrounding gum, but immediately sprung up by refilling of the vessels, or the removal of the pressure. The pain in the tumor was very irregular, both in severity and continuance; some days she scarcely felt it, while on others she had few moments of ease; pressure, however, even with the tongue, was at all times attended with, and followed by pain; the tendency to bleed, too, had been, and still was, a source of great alarm to the patient.

The patient was directed to apply tannin in powder to the surface of the diseased part every three hours. With this treatment the tumor became gradually diminished in size, became less vascular, and finally altogether disappeared.

The second case was similar to the one now narrated, and yielded to similar treatment.

I do not recollect to have seen a description of this disease; neither have I seen any other cases than the two now mentioned.

*Blue Gum.*—Dr. Watson, in his lectures, when treating on colica pictonum, says—"Very recently a most curious symptom, pathognomonic, I believe, of the presence of lead in the system, has been pointed out by Dr. Burton; and, now that it has been pointed out, one can hardly understand how it escaped us so long. It is a blue or purplish line running along the edges of the gums just where they meet the teeth."* A paper on this subject, by Dr. Burton, was read at the Medical and Surgical Society, in January, 1840.

It is not my purpose to say any thing on the general effects of lead on the system; but I shall take this opportunity of relating to you a few observations on blue gum induced by lead, and the conditions necessary to its production. My position at the hospital has enabled me to make many inquiries into this curious phenomenon, which would naturally escape the observation of those whose vocation does not lead them to inspect in large numbers the teeth and gums of healthy subjects.

It has been observed that the presence of teeth is necessary to coloring of the gum. This is not all, however: the necks of the teeth must be encrusted with tartar, otherwise the edge of the gums will not receive the blue tinge: in fact the teeth are necessary only as affording lodgment for the tartar. I conceive, that, if tartar were held in constant contact with the edge of the gums by any other means than its lodgment on teeth, the gums would be equally tinged. I have frequently seen that gums about teeth encrusted with tartar were very blue, and in the same mouth the gums about teeth free from tartar, perfectly natural in color.

The coloring of the gum may be the sole indication of lead in the system. I not unfrequently find among my patients those whose gums are blue, but who declare that they have never suffered from colic or any other effect of lead; and, indeed, a few of these say they have not, to the best of their knowledge, been exposed to lead.

A short time since, a gentleman applied to me to remove a troublesome tooth. I found the necks of the teeth encrusted with tartar, and the edges of the gums intensely blue. He stated, on inquiry, that he had just returned from China, and that during the voyage he had been salivated for syphilis, but that he had not, so far as he knew, been exposed to the action of lead, either by inhalation or any other mode.

* Dr. Watson's Lectures on the Practice of Physic.

The continuance of tartar on the teeth is necessary to the continuance of blue gum. If the whole of the tartar be removed from the neck of the tooth, the blue tinge on the gum will gradually fade, while its intensity will be preserved about the teeth on which the tartar is allowed to remain. I cannot tell you how long it will be before the color will be wholly gone, when the tooth is allowed to remain, because the tartar may reaccumulate, and thus defeat an experiment for that purpose. When the tooth is removed, however, the blue stain disappears in two or three weeks, as the following case would indicate: I was called to remove an aching tooth for a lady who had taken two or three doses of acetate of lead for the suppression of uterine hæmorrhage. The gums exhibited the characteristic blue line. Nine days after the extraction of the tooth, the gums had come together, and the union was marked by a transverse blue line. At the expiration of three weeks the blue line had wholly disappeared.

In another well marked case of blue gum the patient had nothing to do with lead in any way, and had not been in the neighborhood of recently applied paint; but he was employed silvering mirrors, in which mercury and tin are the metals used.

These are not solitary instances in which I have found strongly-marked blue gum, and yet no other indication of the presence of lead in the system, or of the exposure of the patient to the action of lead. Hence I am forced to suspect that other metals may produce a similar discoloration of the gum. Should future investigation prove this suspicion to be well founded, the diagnostic value of this state of gum in relation to lead will be diminished.

In endeavoring to trace by what process the gums are stained blue, it must be borne in mind that the tartar itself is often similarly discolored, especially where it is in contact with the gum. The coloring material is probably sulphuret of lead, or a similar salt of some other metal. Tartar being very porous, admits into its substance fluids charged with animal matter which may there be decomposed, and furnish sulphuretted hydrogen, as a common product of decomposition. Supposing a salt of lead to be present the tissue of an adjoining part, a sulphuret of lead would be formed, which would give the color in question to the tissues in which the formation took place. This action would be continuous so long as the metal remained in the system, and the tartar remained to afford a site for decomposition, and to hold the products against the gum.

The saliva itself contains sulpho-cyanic acid, and from this source sulphur might also be furnished.

Traces of lead may be found in the tartar of those affected by the metal.

*Tartar a Salivary Calculus.*—The saliva, together with oral and pulmonary mucus, hold in solution various salts, which are precipitated in a greater or less quantity on natural or artificial teeth in those situations where the solvent fluids remain at rest. Epithelial scales and other extraneous matters that may be floating in the oral fluids, or entangled between the teeth, become impacted in the precipitated salts, and thus contribute to form the concretion usually called *tartar*. And, in addition to these, infusorial animalcules are met with in recent tartar, and their remains in that which has been dried.

Simon says, "Tartar on the human teeth consists of earthy phosphates, epithelium-scales, a little ptyalin, and fat; and when examined under the microscope there are seen abundance of pavement epithelium and mucous corpuscles: and in addition to these, numerous long acicular bodies and infusoria of the genera vibrio and monas."

According to Berzelius, tartar is composed of—

| | |
|---|---|
| Earthy phosphates, . . . . | 79.0 |
| Salivary mucus, . . . . . | 12.5 |
| Ptyalin, . . . . . | 1.0 |
| Animal matter soluble in hydro-chloric acid, . | 7.5* |

Dr. Wright gives the following as the constituents of healthy saliva:

*Simon's Animal Chemistry, translated by Dr. Day.

| | |
|---|---|
| Water, . . . . . . | 988.1 |
| Ptyalin, . . . . . | 1.8 |
| Fatty acid, . . . . . | .5 |
| Chlorides or sodium and potassium, . | 1.4 |
| Albumen and soda, . . . . . | .9 |
| Phosphate of lime, . . . . | .6 |
| Albuminate of soda, . . . | .8 |
| Lactates of potash and soda, . . . | .7 |
| Sulphocyanide of potassium, . . . | .9 |
| Soda, . . . . . | .5 |
| Mucus with ptyalin, . . . . | 2.6 |

Berzelius gives the following as the constituents of nasal mucus, to which the bronchial mucus is very similar:

| | |
|---|---|
| Water, . . . . . . | 933.7 |
| Mucin, . . . . . | 53.3 |
| Alcohol-extract and alkaline lactates, . . | 3.0 |
| Chloride of sodium and potassium, . | 5.5 |
| Water extract, with traces of albumen and phosphates, | 3.5 |
| Soda combined with mucus, . . . | 3.9 |

Tartar has been described by dentists as consisting of several different kinds, and named from the variation of color and density it presents. Thus, one sort is called black, another green, a third yellow tartar. The division is not, however, so far as I know, based upon any chemical difference, and may, therefore, be disregarded. I conceive that in most instances these physical variations are traceable to the time occupied in the formation or to the habits of the individual.

Thus, when tartar collects quickly, it is usually soft and yellow; and, on the other hand, when the process is slow, it is dark and hard. Then, again, in those who smoke much, the tartar is of a deep brown or black color. In teeth where one fang has been necrosed, and stripped of periosteum, the surface of the dead fang is often studded with modules of very hard greenish tartar. This tartar, during the time of its deposition, has been bathed in pus secreted from the lining membrane of the socket. The tartar is not an active corrosive agent, produccing the destruction of the fang, as some dentists have supposed, but is consequent on its death and denudation.

We commonly find the concretion the most abundant about the posterior surfaces of the front teeth of the lower jaw, or about the buccal surface of the molares of the upper jaw, if from any cause those teeth are not used in mastication.

If a vertical section of a piece of tartar, carefully removed, be made, it will be found to present an inclined plane, the base of which lies in contact with the gum. The surface towards the tongue, or cheek, is usually smooth, but that against the gum is rough; and it is to that additions are mostly made. The gums become irritated and inflamed from the contact of the rough surface of the tartar; the alveoli become absorbed, and the gum recedes, making way for the further accumulation of the salivary salts. To the dental tissues themselves the tartar does no direct injury, but its effect upon the gums and alveoli is destructive, and hence, indirectly, upon the teeth.

Careful daily brushing will do much to prevent the accumulation of tartar on the teeth, but should it accumulate it must be removed from time to time by instruments fitted for the purpose.

Tooth powder that will dissolve the tartar will also dissolve the teeth, and therefore may not be used.

### LECTURE XV.

OPERATIONS ON THE TEETH.—SCALING THE TEETH—PIVOTING OR GRAFTING TEETH—PLUGGING OR STOPPING TEETH—ON THE EXTRACTION OF TEETH—GENERAL RULES TO BE OBSERVED—ON EXTRACTING INCISORES, CANINES, BICUSPIDES, AND MOLARES—THE EXTRACTION OF STUMPS.

### *Operations on the Teeth.*

WE have now arrived at the third division of our subject, namely, to *Operative Dentistry.* I have in previous lectures told you when operations should be had recourse to; I must now tell you how these operations are to be performed. The subject being altogether mechanical, I can only direct you how to proceed, and what, with our present knowledge, constitutes success. I cannot give you dexterity; the attainment of that will depend on your own perseverance and manual aptitude.

*Scaling.*—This operation consists in removing the tartar from the teeth, and is performed by the help of small steel instruments, of various forms, suitable to the parts upon which they are intended to operate. The instrument-makers will furnish you with a great variety of patterns from which to choose: those on the table are the ones I make use of. The point of the instrument should be inserted under the base of the tartar, and by pressing the instrument towards, or rather outwards from, the crown of the tooth, detach the tartar in a mass. Having broken away the bulk of the tartar, each tooth must be freed from the smaller adherent particles, by carefully scraping its surface with the edge of the scaler. The intervals between the teeth must be treated in a similar manner, till every tooth is completely free from the concretion. The teeth may then be well rubbed with a piece of cane, or a brush, or a piece of soft wood, loaded with fine pumice powder, or powdered turkey-stone, and after this again with a little precipitated chalk. By these latter processes the surfaces of the teeth are well polished, by which their appearance is much improved, and their liability to become again encrusted diminished.

The patient should be directed to well brush the teeth, night and morning, with precipitated chalk, flavored to the taste of the user. Should the saliva be acid, (you will learn this by the use of test-paper) then the dentifrice should contain a portion of carbonate of soda: ʒ vij. of chalk and ʒ j. of carbonate of soda form a good alkaline powder.

If the teeth are discolored, patients will often ask you to restore them to their original whiteness. This cannot be done. Acids will for a time improve their appearance, by dissolving the outer surface of the enamel, and thus exposing a new surface, but they soon again become discolored and decay. Nothing can be more mischievous to teeth than the use of acid tooth-powders, neither should any but an impalpable powder be used, otherwise the teeth will be worn into deep grooves, and are ultimately destroyed.

Areca nut charcoal has the effect of making the teeth look very white, and its use need not be objected to: charcoal made from wood should be forbidden, for it contains silex, and will wear the teeth. In choosing a dentifrice, your first care must be to ascertain whether or no the saliva is acid, and is acting upon any of the teeth. And this point must determine whether the polishing powder shall contain a soluble carbonate or no.

Several years since, a notice appeared in the Medical Gazette, stating that sugar acted directly on the teeth. I put some teeth into sugar and water and in honey, but at the end of six months I could not find that they were at all affected. Latterly much has been said against camphor as destructive to the teeth, though, as far as I know, with insufficient reason.

*Plugging, or Stopping Teeth.*—A well-stopped tooth, if the operation has not been too long postponed, is perfectly restored to its former durability and usefulness. I removed last year, from an old man, a molar tooth that had been plugged for thirty years, and had been serviceable till within the last two years, when it became loose from absorption of the socket. You will often see teeth that have been stopped ten and twenty years.

Seeing, then, that so much may be gained by this operation in preserving the teeth, you cannot give too much attention to its practice; for while it is among the most useful, it is the most difficult operation the dentist has to perform. This opertion is divided into two parts; the preparation of the cavity for the reception of the plug, and the insertion of the plug. In the preparation of the cavity two points must be gained, otherwise the subsequent steps of the operation will be ineffective.

The first of these is to completely remove all the softened dentine; the second, to get a firm and regular orifice, of sufficient size as to enable the plug to be inserted, and at the same time not too large. If the cavity be large and the opening small, it will be almost impossible to make the plug solid in those parts of the cavity which are overhung; and, on the other hand, if the opening be large and the cavity small and rounded at the bottom, like a saucer, the plug will not be retained. The best form of cavity has a circular orifice with perpendicular walls; in fact, cylindrical.

The situation of the disease must regulate our manner of proceeding. If the cavity be situated in the opposed side of a molar, the tooth must be cut away with a sculper or graver till an excavating instrument can be used. If the sides of the front teeth are affected, a piece of vulcanized caoutchouc should be straightened tight, and then introduced between the teeth; this, in endeavoring to regain its former figure, will separate the teeth sufficiently for the operator. When the masticating surface of a tooth is carious there is no difficulty in the operation; if the extent of the disease be slight, it may be removed by a broach of proper size. Having reduced the cavity, as nearly as attainable to the conditions I have described, the chips must be washed out and the cavity wiped dry with cotton-wool, and the plug inserted. In making the plug, our aim must be to so perfectly fill the cavity that all moisture shall be excluded, and that it shall be sufficiently hard to resist, equally with the tooth the wear of mastication. Unless these two conditions are fulfilled, our work will be imperfect, and ultimately fail.

Gold or tin foil are the best materials for making plugs. Whichever of these be chosen, the method of use is the same.

There are three methods of introducing foil for making a plug. In one the metal is folded into narrow strips, proportioned in width and thickness to the size of the cavity. One end of the strip is, by means of a conveniently shaped stopping instrument, pressed to the bottom of the cavity. The strip is then bent, and a fold passed to the bottom of the hole, leaving the first fold projecting above the surface. Fold after fold is introduced till the cavity is tolerably full. A wedge-shaped instrument is then introduced, and the gold pressed towards the walls of the cavity; more gold is, by a similar process, pressed into the cavity so obtained. This process is repeated till the wedge cannot be forced into the plug. A flattened instrument is then used to compress the gold in the cavity. When we can make no further effect on the surface of the plug by compression, the surface is filed smooth and burnished. By a careful adherence to this plan, we make a plug composed of layers of metal arranged parallel to the walls of the cavity, and therefore not liable to fall to pieces or come out. But, on the other hand, had we made the folds at a right angle to the walls, and parallel to the bottom of the cavity, layer after layer would have peeled off till little or none of the plug remained, and the decay would have proceeded to destroy the tooth.

In the second method, a piece of foil of sufficient size is rolled hard, and spherical between the thumb and finger. This is gradually forced into the cavity, care being taken to get it well in round the outer walls. When the plug has been rendered as solid as possible, the superfluous portion is cut or filed off, and the surface burnished.

The third method of using metallic foil is a combination of the two preceding ones. A piece of foil is rolled up loosely that will readily go into the cavity. When in its place a wedge-shape instrument is passed into its centre, which has the effect of spreading the gold towards the walls of the cavity. The centre is gradually filled with folds of gold in the manner I have described. The wedge is used again and again till it can no longer be made to enter. The gold is then compressed on the surface, and the superfluous portions removed, and the surface burnished. When the plug is finished in either of the manners I have described, the circumference should be examined by a sharp steel probe. If this can be made to enter at any part, the hole so made should be enlarged by thrusting in an instrument as large as can be introduced, and the hole filled.

Either of the foregoing methods of plugging will answer, if well done. But of these I prefer introducing the metal in folds. The situation of the cavity, and also the size, will have something to do with the selection of the plan of operating. Then, again, one person will be more apt at one manner of procedure than at another. All these matters of detail must be learned in practice. I should exhaust your patience, and greatly exceed my limits, were I to attempt to describe every variety in form and situation of cavity, and every modification and plan useful in plugging.

Where the cavity of a tooth is so large that the walls are too thin to bear the pressure necessary to the insertion of a gold or tin-foil plug, the amalgam of silver or of palladium may be advantageously used. Having prepared the cavity as for the use of foil, a little mercury is triturated in a glass mortar with a small quantity of precipitated silver or palladium, till they unite and form a paste, which is well squeezed in a piece of wash leather to force out as much as possible of the mercury. The paste is then again rubbed in the mortar, or in the palm of the hand, and then introduced into the cavity. The cavity, however, must be first well dried with lint, and care must be taken to get the amalgam in close contact with the whole circumference of the cavity.

The plug so formed hardens in the course of a few hours, after which the surface should be well burnished. The American dentists condemn this kind of plug, as it seems to me, somewhat unjustly. It is undoubtedly far inferior to either the gold or tin-foil plug, but it can be used where they cannot, and it is surely better than none. I have seen a mere shell of a tooth, that would have broken away on the first attempt at introducing foil, rendered useful for years by an amalgam plug.

Before leaving the subject let me warn you that unless the cavity be well prepared by the total removal of the softened dentine from the walls, and by getting a good, firm, and well-shaped orifice, free from acute angles, no plug will answer, and least of all the amalgam. It will fall out or become loose within twelve or eighteen months, and frequently in much less time, and decay will proceed. Teeth plugged with silver amalgam usually become stained of a deep blue-black color. When the palladium amalgam is used there is little or no staining, if the excavating be perfect. The latter amalgam is therefore preferable.

*The Operation of Pivoting or Grafting.*—When the crown of a single-fanged tooth has so far decayed that it cannot be plugged, and yet the root remains sound, a new crown may be fastened into the root by means of a piece of gold wire called a pivot: hence the name pivoting.

The operation is commenced by sawing or cutting off the crown of the faulty tooth. The pulp is then destroyed by passing up the cavity of the tooth a fine broach, and giving it a turn or two, by which the pulp is torn across near the extremity of the fang. The surface is then filed with a half-round file even with the edge of the gum. The pulp cavity is then enlarged and made cylindrical by a drill or broach similar in size to the wire we propose using for the pivot.

We must now select a tooth, whether mineral or natural, corresponding in size, shape, and color to the one lost, and from this the root must be removed, and the crown fitted accurately, by filing, to the surface of the fang to which it is to be attached.

A hole must now be drilled into the fitted crown, and a screw cut in it; a screw must also be cut upon the wire, which must be screwed into the crown, and a sufficient length left projecting from the crown, to occupy the hole drilled in the fang. The projecting part of the pivot is rendered a little rough, to retain a very thin coating of floss silk. In this condition it is passed into the fang, and if the adjustment has been carefully performed, the new crown will fit so accurately to the fang that the artifice cannot be detected.

The Americans sometimes use compressed wood instead of gold wire for the pivot. You will sometimes find it very convenient to adopt this plan in renewing a pivot, or when the hole in the stump is necessarily rather large.

Pivoting, though the neatest, is more frequently followed by mischievous results than any other operation performed by the dentist, and so common are these that many consider the operation as unjustifiable. Some degree of pain and tenderness is almost always felt during the first few days after the operation, and unfortunately it is not uncommon to have considerable inflammation ending in suppuration, as in the case I cited to you in Lecture XIII. But the consequence may be even worse than in that case.

The following statement was placed in my hands by a medical man who had some knowledge of the case which is related. ———, Esq. aged 25 years, tall and thin, but apparently in very good health. On his marriage trip he visited Paris, and there had the misfortune to break off a front tooth. Wishing to conceal the accident from his wife, he went immediately to a dentist. The tooth was pivoted (and I have no doubt carefully, for the dentist was one with a great and just reputation,) and the necessary concealment seemed insured. From the time of the operation, however, he had severe pain in the stump, which pain increased for four or five days, when he left Paris for Rouen. Upon arriving there the pain had become excessively severe; he consulted a medical man, but it was too late. Trismus came on within twenty-four hours, and was soon followed by tetanus and death.

*On the Extraction of Teeth.*—This is not the first time I have treated on the extraction of teeth. In 1841* I described and figured, with some pleasure, what I believed to be new and much improved forms of tooth-forceps, as possessing great advantages over the key instrument, then almost universally used. Now, after the lapse of six years, I am again on the same subject, and I shall proceed with increased pleasure; for during the interval the instruments in question have come into general use. To borrow an instrument-maker's phrase, "no other tooth instruments will sell now;" whereas, previous to the date of my paper, they had no such instruments to offer for sale.

Nay, in further testimony of the value of the improved forceps, a member of my own profession, after ordering a set of my instrument-maker, found them, on trial, so serviceable, that he was induced to write a little work minutely describing them, and strongly recommending their general adoption to the exclusion of the instruments then in use;† though, for the honor

* Medical Gazette, June 4th, 1841.

† Observations on the Extraction of Teeth, by T. Chitty Clendon, 1843.

and good name of our profession, I regret to say he altogether neglected to acknowledge by whom the instruments of which he had availed himself, and was describing, were designed and brought to their present state of utility, and published. Indeed, so anxious has this author been to take to himself whatever credit there may be in the improvements, that he has, whenever he had power, induced the makers to stamp the instruments with his name.

It is enough, however, that our means of operating have been improved, and that this improvement, whereby suffering is diminished, has been generally adopted. It matters not to the patient who invented or improved, or gave to the profession the instrument with which his tooth is removed, so long as that instrument is of the best kind, and skilfully used.

A few dentists have for many years been in the habit of purchasing their tooth-instruments in the rough, and filing them up into whatever shape they deemed best, but what this shape was, or is, was kept secret. Indeed, those who purchased finished instruments very commonly ordered the maker not to make known any of the particulars of construction. This time of professional secrecy has passed, or is fast passing away, and much to the credit of our profession.

I shall not have space to give you a history of tooth-drawing, and of the various instruments at different times in use, but must content myself with describing, as briefly as possible, the means and methods of operating we now employ.

In extracting a tooth, the following conditions should be fulfilled: First. The whole of the offending organ should be removed.

Secondly. It should be removed with as little injury as possible to the structures in which it is implanted.

Thirdly. The patient should be spared all unnecessary pain in the operation.

That method by which a tooth, or the remains of one, can be removed most certainly, quickly, and at the same time with the least amount of injury to the adjoining parts, will also remove it with the least pain; the suffering being in most cases proportioned to the duration of the operation.

To fulfil these indications, recourse must be had to an instrument so formed that it shall grasp the tooth alone, and by the required force applied nearly in the axis of the tooth, remove it. Such instruments are forceps; but forceps so constructed that they shall accurately fit the tooth to be extracted, and so fashioned at the jaws, nibs, or blades, that they shall readily separate the gum from the neck of the tooth, to which point they shall arrive by simply placing the extremities of the jaws at the edge of the gum, closing the handles, and at the same time pressing the instrument firmly and steadily in the direction of the tooth, till it comes in contact with the free edge of the alveolar process.

As the teeth are variously shaped, so will it be necessary to have forceps of different forms; in fact, a pair fitted to each kind of tooth. By forceps so constructed most teeth may be removed in less time than by any other tooth-extracting instrument at present in use; also with less pain to the patient, and without inflicting any farther injury to the gums and alveolar processes, than must necessarily result from the forcible separation of a tooth from its natural attachments.

An instrument which in its employment requires that force should be applied to the contiguous parts as well as to the tooth to be extracted, is an imperfect instrument for the required purpose; or an instrument which, by its form and mode of application, requires that greater force should be used than would be necessary for the dislodgment of the tooth, supposing that force to be applied in the most advantageous direction, is also an imperfect instrument, and but ill fitted for the purpose for which it was designed. Such, however, are the imperfections of an instrument till recently in very general use for the extraction of teeth—the "key;" in the application of which the fulcrum is rested on the alveolar process, between which and the fulcrum the interposed gum is subjected to considerable pressure: just as much, indeed, as may be necessary for the dislocation of the tooth. By this treatment the gum is often considerably bruised, sometimes so much so as to lead to suppuration of the injured part, and always to an unpleasant degree of soreness. The force used in extracting a tooth with the key must be much greater than the actual force required to effect the operation, because the power is applied solely in a lateral direction—a direction in which the tooth offers great resistance, especially in molares of the lower jaw, where the lateral alveoli are strong, and composed of dense bone.

There are several specimens on the table of two and three teeth with their alveoli torn away from the jaw, in attempting to extract a single tooth with the key. These accidents could not have occurred had forceps been used.

I am quite aware that there are many and eminent surgeon-dentists who use and speak very highly of the "key" instrument; and no doubt it is very much to be preferred to the forceps which were ordinarily kept by surgical instrument makers: forceps applicable to no one tooth in particular, when applied touch only at two or three points, and are quite incapable of being passed down to the neck of the tooth, unless the gum be previously cut away, and even then very imperfectly.

Dr. Flagg of Boston, has contrived forceps for the extraction of every kind of tooth. I have not been able to get a sight of any of his instruments, but, from the description and plates given of them in Fitche's "System of Dental Surgery," I believe they are unlike those on the table.

A work entitled "Operations on the Teeth," by Mr. Snell, contains a description, with plates, of a form of forceps used by the author for the extraction of the molares. The principal aim of the inventor was to have two points of metal coming out from the edge of each jaw of the instrument; the object of these points being to be passed between the fangs of the teeth. These forceps admit of but very limited use, as the fangs of the molares, especially the second and third, are likely to be agglutinated; which condition, in each particular instance, is known only after the tooth has been extracted: but besides this, it is a matter of some difficulty, in any case, to get these two points between the fangs; and thereby inflicts unnecessary pain, supposing the tooth could have been removed without this violence.

In those instruments a point was made to project from the middle of the edge of the jaws, which, on grasping the tooth, should pass under the body of the crown and between the fangs. The forceps were unfitted for, and did not come into general use on two grounds: 1st. The fangs of the second and third molares are often united, in which case the point or points would be worse than useless; and we cannot know for certain the state of the fangs till the tooth has been removed. 2d. The insertion of the points is always attended with greater pain than results from seizing a tooth with adjusted forceps; and, excepting some cases which I shall afterwards describe, is attended with no compensating advantage.

All forceps whatever should embrace the tooth they are used to extract at its neck; the neck being situated between the termination of the enamel and the free edge of the alveoli, and covered by gum. In order to arrive at this part of the tooth without difficulty, or unnecessary pain to the patient, the jaws must present an inclined plane, terminating in an edge. The external surface of the jaws of the forceps, when closed, should present something like a cone, or parts of several cones, with the apex or apices cut off; and a perpendicular section should present an inclined plane, terminating in an edge, but more or less curved, as may be suitable to the particular instrument. The length from the joint to the edge of the jaws should on no account be greater than will be necessary to allow sufficient space for the reception of the crown and neck of the tooth, so that no strength may be lost.

An average tooth should be selected and given to the forceps-maker, and he should be instructed to make the jaws fit the neck of the tooth exactly, leaving sufficient room for the crown of the tooth to be free from pressure, but not more than will be necessary to clear the enamel.

The fangs of all teeth having a general conical form, forceps, when well made and applied, should be but as a lengthening of the cone towards its base. For removing teeth which are not decayed down to the gums, the ends of the jaws should be square or slightly rounded; but when nothing but the fang remains of a tooth, rounded ends are the more convenient, as with that shape they are readily introduced between the fang and inclosing alveolus. Instruments for extracting stumps should be made altogether lighter, the jaws thin and sharp at their edges, so that they may be made to cut rather than tear the membrane connecting the fang with the adjoining tissues. But I shall say more on the extraction of stumps presently.

Forceps should be constructed and used upon the principle of lengthening the tooth for the extraction of which they are intended: thus enabling the operator to move it from side to side, or rotate it if the fang be single, and of a shape admitting of such motion.

After these lateral movements for destroying the membraneous union have been effected, the tooth may, unless the fangs have some peculiair position or shape, be raised in a perpendicular direction, leaving as little injury from its removal as the operation can admit.

When forceps are used for the extraction of teeth, the operation is divided into three stages—1st. The seizure of the tooth; 2d. The destruction of its membraneous connection with the socket; 3d. The removal of the tooth from the socket. When you commence operating, it will be of great service to you, and advantage to those operated on, that you should pay strict attention to these stages, and that each should be well and efficiently executed before you proceed to its successor; for, should the tooth be unskilfully seized, the crown will be broken off in the attempt to detach the tooth from the periosteum of the socket, until which is effected, the fangs cannot be removed from their bony cells. You will find that a tooth will resist a great force applied in a line with its axis, or, in other words, if you attempt to pull a tooth straight from its socket.

In seizing a tooth, the jaws should be closed lightly on the tooth, and inserted under the free edge of the gum, and then *forcibly* driven down to the edge of the alveoli, or even a short distance into them. I say forcibly, because all beginners, and even some practised in the use of forceps, are liable to failure because they do not use sufficient force: they seize the tooth at the tooth at the edge of the gum, instead of at the edge of the alveoli.

I wish to impress upon you the absolute necessity of laying hold of the tooth as far down towards the fangs as you can possibly get the instrument. An old and successful operator, when instructing another in the use of forceps, said—"Push them into the sockets as though you intended they should come out at the top of the head, or under the chin."

The way of effecting the second stage will depend on the shape of the tooth to be removed; as also will the third, and to this we will now address ourselves. But different shaped teeth require forceps shaped to them. It will be necessary, therefore, to describe partially the teeth, and then the forceps individually, that the peculiar shape of each instrument may be understood. Before doing so, however, I should state that it is quite impossible for any person to extract teeth properly, whatever instrument may be used, especially if the forceps be chosen, unless the operator is perfectly acquainted with the form of each tooth, with the relative position and size of the fangs, with their direction in the alveoli, with the general form of the alveoli themselves, and with the directions in which they offer the greatest and the least resistance.

